# Prevention and Detection of Mycoplasma Contamination in Cell Culture

**Published:** 2011-12-22

**Authors:** Laleh Nikfarjam, Parvaneh Farzaneh

**Affiliations:** Human and Animal Cell Bank, Iranian Biological Resource Center, ACECR, Tehran, Iran

**Keywords:** Mycoplasma, Cell Culture, Prevention, Elimination

## Abstract

One of the main problems in cell culture is mycoplasma infection. It can extensively affect cell physiology and metabolism. As the applications of cell culture increase in research, industrial production and cell therapy, more concerns about mycoplasma contamination and detection will arise. This review will provide valuable information about: 1. the ways in which cells are contaminated and the frequency and source of mycoplasma species in cell culture; 2. the ways to prevent mycoplasma contamination in cell culture; 3. the importance of mycoplasma tests in cell culture; 4. different methods to identify mycoplasma contamination; 5. the consequences of mycoplasma contamination in cell culture and 6. available methods to eliminate mycoplasma contamination. Awareness about the sources of mycoplasma and pursuing aseptic techniques in cell culture along with reliable detection methods of mycoplasma contamination can provide an appropriate situation to prevent mycoplasma contamination in cell culture.

## Introduction

These days, the application of cells in research laboratories (1, 2), regenerative medicine (3, 4) and biotechnological productions is growing extensively. Cells are used in wide-ranging activities from studies on cell proliferation to the production of biologically active substances. Due to restrictions on the use of laboratory animals by animal protection laws, the use of cell cultures will continue to increase in the future. In order to achieve reproducible results from cells, good cell culture conditions are vital. Despite the importance of bacterial and fungal contaminations in cell culture, they are not such a serious problem because they are usually obvious and easily detected. The most serious problem is mycoplasma infection since these microorganisms are subtle (5, 6).

Mycoplasma has generated considerable interest due to its ability to contaminate cell lines used in research as well as in manufacturing bioproducts (7-13). The lack of a cell wall in mycoplasmas besides their adherence to the cell surface makes them invisible to the naked eye. In the past, the use of animal sera in cell cultures was the main source of Mycoplasma arginini, Mycoplasma *hyorhinis* or Acholaeplasma *laidlawii*. Pipetting through mouth is the source of Mycoplasma *orale*, Mycoplasma *fermentans*, Mycoplasma *salivarium* and Mycoplasma pirum (14, 15). Culture supernatants and cell membranes are suitable for the growth of mycoplasma. Mycoplasmas are resistant to commonly used antibiotics and they cannot be detected visually by turbidity of fluid or under the inverted microscope. The frequency and impact of mycoplasma contamination in cell culture have been extensively discussed (12, 15-17). The incidence of single mycoplasma contamination is still high, being 15 to 35% worldwide with extreme incidences of 65 to 80%, whereas incidences of multiple mycoplasma infections with two or more mycoplasma species are between 7 and 60% (9, 10, 18, 19). Mycoplasma contamination can be persistent and difficult to detect for the affected lab (10). Between 5 and 35% of cell cultures are infected with the species Acholeplasma *laidlawii*, M. arginini, M. *fermentans*, M. *hyorhinis* and M. *orale* (12). It is estimated that about 5 to 30% of the world's cell lines are contaminated with mycoplasmas [5-16%, (14); 5-87%, (15); 25.7% (20); 29% (21) and 23% (22)]. Mycoplasmal contamination influences almost every parameter within the cell culture system (8, 23).

 The use of contaminated cells endangers almost all aspects of cell physiology, and often leads to erroneous results or causes the loss of unique cell lines (24, 25). Even though the mycoplasma contamination does not slow down cell metabolism, it may contaminate the final product (such as a vaccine) resulting in the loss of the batch (26). Mycoplasmal infection of cell cultures might often linger for an extended period of time without noticeable cell damage (7, 23, 27). Therefore, it is important to use efficient detection methods to inspect mycoplasma contamination (12, 5, 28, 29). Usually, investigation of mycoplasma is carried out by direct and indirect detection methods (30). The direct method detects the colony growth of mycoplasma on agar; however, the indirect detection technique consists of measuring a gene product that is linked to mycoplasmas rather than to the mammalian cells in culture. Moreover, DNA staining of mycoplasma after inoculation of suspected cell onto indicator cells is another type of indirect detection method of mycoplasma (31, 13). 

The first source of contamination is usually contaminated media or their components (7, 32). Most cell culture media are not autoclavable; therefore, filtration of the media through proper filters to remove mycoplasma is very important to protect cell culture lines. The prevention of mycoplasma contamination can be divided into three categories: cell culture facility, cell culture procedures, and operator technique (33). To prevent mycoplasma effectively, it is recommended that a good aseptic technique be used, accidents in the laboratory be reduced, the laboratory be kept clean, and the positive cultures be discarded (34- 37). In the case of valuable and unique cultures, it is possible to eliminate the contaminants effectively. Therefore, treatment of mycoplasma-positive cell cultures has become a feasible option (18, 27).

### What are mycoplasmas?

The name of mycoplasma was chosen because of its mycelated fungi-like structure with a flowering plasma-like structure (38, 39). Mycoplasma is a kind of bacteria. One of the differences between mycoplasma and the other bacteria is the absence of cell wall and their flexible membrane in mycoplasma which results in taking different shapes and consequently difficulties in identifying even under a high powered electron microscope. The small size of mycoplasma (0.15-0.3µm) is the main reason for their escape through filtering systems and also their growth in high concentration in mammalian cell cultures without any turbidity or other obvious symptoms (40). However, there is some exception in the size of mollicutes. Recently it has been shown that different nutritional conditions can affect the size of Acholeplasma laidlawii. Folmsbee et al. showed Acholeplasma laidlawii cultures in tryptic soy broth (TSB) cannot penetrate 0.2µm rated filters to the same degree in other media such as mycoplasma broth or TSB supplemented with 10% horse serum (41). 

### Ways in which cells are contaminated by mycoplasma

Mycoplasmas can bind to their host cells using special tip organelles. These tip organelles have a high concentration of adhesins, to attach to eukaryotic cells and penetrate the host cell. The lack of a stiff wall in mycoplasma may help it to fuse with the membrane of the host cell and exchange its membrane and cytoplasmic components (38, 42, 43).

### Frequency and sources of mycoplasma species

**Fig 1:**
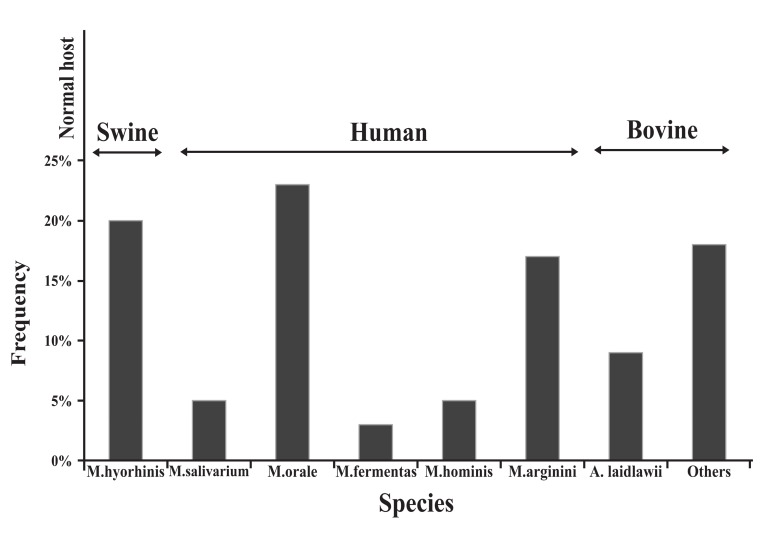
Frequency of different species of mycoplasma occurring in cell culture

There are a number of different sources for mycoplasma contamination in cell cultures associated with human, bovine and swine species. Personnel in the laboratories are the main sources of M. *orale*, M. *fermentans*, and M. *hominis*. These species of mycoplasmas account for more than half of all mycoplasma infections in cell cultures and physiologically are found in the human oropharyngeal tract (44). M. arginini and A. *laidlawii* are two other mycoplasmas contaminating cell cultures and originate in fetal bovine serum (FBS) or newborn bovine serum (NBS). Trypsin solutions provided by swines are a major source of M. *hyorhinis* (45). Figure 1 is a diagram showing the normal host and frequency of different species of mycoplasma occurring in cell culture.

### Different sources for the spreading of mycoplasma in the laboratory

McGarrity designed a model to find out how mycoplasmas spread in a laminar flow hood during a routine subculturing procedure. He intentionally infected a cell culture with mycoplasma. After trypsinization of the infected culture in a laminar flow hood, live mycoplasmas were isolated by the technician, outside of the flask, a hemocytometer, the pipettor, and outside of the pipette discard pan. Live mycoplasma could be successfully recovered from the surface of the laminar flow hood even *four* to *six* days later! A clean culture, that was subcultured once a week in the same hood following the work with the contaminated cells, tested positive for mycoplasma after only 6 weeks. These results show how quickly and easily mycoplasma can spread and also warn us against the possibility of contamination of most if not all of the other cultures after the entry of a single mycoplasma infected culture into the laboratory (46).

Currently, the major source of mycoplasma contamination is infected cultures obtained from other research laboratories or commercial suppliers. Some of the major sources of mycoplasma contamination are listed below:

### Media, sera or reagents contaminated with mycoplasma

Mycoplasmas can pass into the filter membranes used in sterilizing cell culture media, sera and other reagents since they are too small and pliable due to the absence of a cell wall. Therefore, cell culture media and animal products used in cell culture should be considered major routes for mycoplasma contamination (7, 47). In the 1960s and 1970s, sera products were a very important primary source of infection, with reported contamination rates of 18% to 40% (45). Today, sera and media obtained from reputable manufacturers are rarely the source of mycoplasma contamination (13). However, it is still the responsibility of the end user to verify that the products they purchase have been adequately filtered, tested and certified as mycoplasma-free (48).

It is common in most cell culture laboratories to use single 0.2µm pore size filter membranes to filter media or other solutions. However, this method is relatively safe for solutions with low levels of mycoplasma. It is not recommended to filter raw animal-derived sera or products since the mycoplasma contamination could potentially be high in them. To remove mycoplasmas with filtration, the method of filtering plays an important role. Low pressure differential (5-10 psi) is less likely to force mycoplasma through a membrane than filter systems using 20 psi or higher pressure. Filters with 0.1µm pore size should be used instead of 0.2 µm ones in the case of dubious conditions (49).

### Nonsterile supplies, media and solutions

Improper sterilization is a major source of biological contaminants. Packing too much into an autoclave or dry heat oven will cause uneven heating, resulting in pockets of nonsterile supplies. Using too short a sterilization cycle, especially for autoclaving volumes of liquids greater than 500 ml per vessel or solutions containing solids or viscous materials such as agar or starches are other mistakes resulting in incorrect sterilization. To accomplish sterility, the size, mass, nature and volume of the materials for sterilization have to always be considered (50, 51).

Storing sterilized supplies and solutions in a dust- and insect-free area is an obligation to prevent recontamination. Good aseptic technique is also crucial (37, 4848).

### Laboratory personnel

Laboratory personnel are considered a major source of mycoplasma contamination (44). Table 1 shows potential sources of cell culture contamination. M. *orale*, a species commonly found colonizing the human oral cavity and oropharynx, has been the leading contaminant in study after study. Two other human mycoplasma species, M. *fermentans* and M. *salivarium*, are also detected in contaminated cultures but at a much lower rate. Table 2 shows major mycoplasma species found in cell culture and also some of the research results reporting the percentage of contamination with different types of mycoplasma in previous years (40). Table 1: Potential sources of cell culture contamination

**Table 1:** Potential sources of cell culture contamination


Human	Foot	100-1000 organisms/cm^2^
	Scalp	106 organisms/cm^2^
	Forehead	105 organisms/cm^2^
	Sneeze	104-105 organisms
	Saliva	107 organisms/ml
Sterile clothing	After 6 hours	1-6 organisms/cm^2^
Air	Outdoor	100-500 organisms/m^3^
	Indoor	500-2000 organisms/m^3^


In 1976, the role of laboratory technicians in mycoplasma contamination in cell culture was proved. It was shown that the majority (80.6%) of technicians were carriers of mycoplasma, primarily M. *salivarium*. The modes of spreading mycoplasma were evaluated by collecting aerosols generated via talking and sneezing from known mycoplasmal carriers on culture plates. M. *salivarium* can be transmitted during talking and sneezing of technicians in 6.2% and 37.5%, respectively (46).

Street clothes and dirty lab coats are the major source of dust and aerosols. Negligence in wearing a clean lab coat and gloves is a major cause for spreading particles during routine cell culture processing. Furthermore, talking and sneezing also generate a significant amount of aerosols (39, 45). It is highly recommended to avoid working without gloves since frequent hand washing can cause dry and flaky skin which is one of the main sources of particles (52). 

**Table 2:** Major mycoplasma species found in cell cultures and their likely sources


Species	Source of origin	1958- 1972	1966- 1982	1973- 1979	1988	2002
Mycoplasma *hyorhinis*	Swine	15.9%	30.1%	2.0%	26%	10-40%
Mycoplasma *arginini*	Bovine	21.4%	24.8%	2.3%	21%	20-30%
Acholeplasma *laidlawii*	Bovine	8.5%	9.7%	20.0%	5%	5-20%
Mycoplasma *hominis*	Swine	6.1%	2.4%	1.8%	---	10-20%
Mycoplasma *salivarium*	Human	---	<1%	16.2%	---	---
Mycoplasma *orale*	Human	38.8%	34.4%	41.3%	34%	20-40%
Mycoplasma *fermentans*	Human	0.36%	4.1%	1.3%	13%	10-20%
Unidentified *species*			1.2%	15.1%		


**Table 3:** Effects of mycoplasma contaminations on cell cultures


Increased sensitivity to apoptosis
Chromosomal aberrations
Change of gene expression patterns
Changes in cell membrane antigenicity
Inhibition of cell growth
DNA fragmentation due to mycoplasma nucleases
Compromised production of viruses
Inhibition of cell metabolism
Reduction of transfection efficiencies
Cell death


### Incubators

Incubators equipped with fans and air currents are another route for spreading mycoplasma-containing particles during closing and opening of the internal door of the incubator. “Good laboratory practices” are essential to avoid diffusion of mycoplasmas in the incubator and other laboratory devices such as the pipetman, pipet aid and laminar flow. After droplet dispersion in an incubator, bacteria are spread by aerosols (34- 37).

### Liquid Nitrogen

Liquid nitrogen is another cause for spreading mycoplasmas. It is significant that mycoplasmas can survive in liquid nitrogen even without cryopreservation. While mycoplasmas do not proliferate in liquid nitrogen, they are able to contaminate cell cultures stored in liquid nitrogen. Therefore, storing cryovials in the vapor phase of nitrogen tanks is highly recommended (53).

### Airborne particles and aerosols

Airborne particles and aerosols generated during culture manipulations are the greatest sources of microbial contamination. The diameter of microbeladen particles is generally 4 to 28µm and they settle at a rate of almost one foot per minute in still air. As a result, the air in a sealed, draft-free room or laboratory is nearly free of biological contaminants. However, as soon as people enter the room, particles that have settled down will be easily resuspended. Some equipment and activities such as pipetting devices, vacuum pumps and aspirators, centrifuges, blenders, sonicators, and heat sources such as radiators, ovens, refrigerators and freezers generate microbial particles and aerosols. Another source of particles and aerosols is experimental animals whose house and care facilities should be kept as far from cell culture area as possible (54).

### Overuse of antibiotics

It is a common practice in research laboratories to use antibiotics in cell culture to avoid microbial contamination. The consequence of overuse of antibiotics is concealment of the poor aseptic technique and it is a major cause for mycoplasma contaminated cultures. Overuse of antibiotics can also lead to antibiotic resistance. Veterans of cell culture insist on doing cell culture without antibiotics to avoid the mentioned problems (48).

### Improper sealing of culture dishes

Another way of entering microbial contamination in flasks, plates and dishes is improper seal of culture dishes. The route for microbial contamination is provided when the top and bottom sidewalls of dishes or flasks and their caps become wet and microbes transfer by capillary action of the wet surface (46).

### Other mycoplasma contaminated cell cultures

A mycoplasma-infected cell culture is a major source of mycoplasma contamination of other cell cultures in the lab. To avoid mycoplasma contamination in cell cultures, it is recommended to test the new cell lines which are obtained from an outside source. A single mycoplasma contaminated cell culture is enough to endanger other cell cultures in the lab. The contamination can spread by means of aerosols and particulates generated during the handling of the mycoplasma infected cell culture. So, working with only one cell culture at a time and preparing separate media and reagents for each individual cell line can avert mycoplasma contamination (39, 55).

A good cell culture practice and regular testing of all new cell cultures can decrease the risk of mycoplasma contamination (48, 36).

### Methods for prevention of mycoplasma contamination in cell culture

#### Improve aseptic techniques and practices

A precise supervision of new workers in the lab by a skilled operator to follow good aseptic techniques can help to reduce the risk of mycoplasma contamination. It is highly recommended to prepare the reports of all cell contamination incidents to solve the problem of contamination (9).

#### Test cultures for contamination

Since one of the main sources of mycoplasma is the cell cultures brought from outside, it is suggested to supply cells from reliable cell banks.

In the case of the existence of mycoplasma contaminated cell culture in quarantine and the absence of separate incubators, only flasks in a plastic box with lid should be used. Never use plates and unsealed dishes in quarantine. In the case of suspected cultures, handling them at the end of the workday after all other cell culture work is completed, using separated media and reagents, and finally disinfecting the laminar flow hood after working is strongly suggested (9).

#### Only use antibiotics responsibly

Although ideally antibiotics used for cell culture should eradicate all contaminants, be nontoxic for the host cells and not interfere with experiments, none of the available antibiotics meet the mentioned criteria (56). Therefore, application of antibiotics in cell culture should be limited. Instead, a good aseptic practice plays an important role in prevention of contamination.

Microbial contamination in cell culture which is antibiotic free is detectable by turbidity or color changes in cell culture medium. In the case of using antibiotics in a cell culture, there are four possibilities: 1. susceptibility to antibiotics, 2. resistance to antibiotics, 3. partial resistance to antibiotics, 4. resistance to antibiotics only by mycoplasma. The last one is the worst contamination since mycoplasmas can be spread by aerosols. In the case of antibiotic susceptibility, antibiotics prevent the cultivation of bacteria and fungi, but are incapable of precluding mycoplasma from the beginning. Therefore, continuous use of antibiotics for a long time in cell cultures not only is not helpful, but also can cause more problems. However, the use of antibiotics (Penicillin/ Streptomycin) for a short term (the first two weeks) in primary culture is vital. Since antibiotics are unstable in the medium, it is highly suggested to replace antibiotic containing medium with fresh medium every two or three days (57, 40).

#### Discard or treat mycoplasma contaminated cells

In the case of mycoplasma contamination of cells which are really valuable and rare, it is suggested to treat them to eliminate mycoplasma infection. Otherwise, it is recommended to discard mycoplasma contaminated cells since they are considered as a source of contamination in the lab (58).

#### Quarantine new cells of any origin

As described in previous sections, new cells which are brought from other laboratories are characterized as a main source of contamination. So, it is necessary to quarantine cells and check for mycoplasma contamination before using them for any purposes (25).

#### Reduce aerosol generation

Aerosols during manipulation of cells in the laminar hood or in the incubators and also aerosols made by personnel can transfer mycoplasma contamination in the lab. Therefore, avoiding activities which result in making aerosols can help to prevent mycoplasma contamination in cell cultures (59).

#### Importance of mycoplasma tests

Mycoplasma contamination rate in cells which are in use or are banked in cell banks in USA as well as in Europe is about 15 to 35%, according to reports (9, 10, 18, 19).

As mentioned previously, the main sources of mycoplasma contamination in a cell culture laboratory are animal-derived media products, laboratory personnel and cross contamination of other contaminated cell lines. It is common to have up to 107 mycoplasma with human, bovine or porcine origin per milliliter of cell culture supernatants, but the appearance and the behavior of cell cultures are quite normal. Mycoplasmas cannot be seen by visual examination or light microscopy. Accordingly, experiments in which cell culture is used are at risk and their results cannot be reliable without checking for mycoplasma contamination. Moreover, nutrient competition or toxic metabolites due to mycoplasma can affect bioproducts which are produced by mycoplasma contaminated cell cultures.

Practically, elimination of mycoplasma is almost impossible with antibiotics or even the newly developed "mycoplasma elimination reagents". Although several published reports claim that a complete decontamination and curing is possible, the mycoplasma experts at Institute of Bacteriology, Mycology and Hygiene (IBMH) have proved that some mycoplasma species can escape from elimination procedures and invade cultured eukaryotic cells. This is due to the mechanism of internalized mycoplasmas that leave the cells and contaminate new cells (60, 61).

Elimination of mycoplasma is very difficult, if not impossible, because some mycoplasmas hide. So, the easiest way to avoid mycoplasma contamination in cell cultures is to examine them periodically. A routine mycoplasma examination can reduce the hazard of concealed mycoplasmas in cell cultures. By excluding the positive samples, any serious problem will be avoided. In academic research, the quality control of mycoplasma contamination is still underdeveloped. Regular quality control and biosafety testing are as important as high GMP standards in biotechnological and biopharmaceutical industries.

#### Identification methods of mycoplasma contamination in cell culture

The microbiological culture method is one of the best methods and also an officially approved method to detect mycoplasma contamination in cell culture (62). In this test a sample of cell culture supernatant is added to a liquid medium for mycoplasma culture. After few days, a sample of cell culture supernatant is cultivated on a mycoplasma agar medium and will incubate in an aerobic condition at 37ºC for two weeks. Positive samples on agar plates will show small colonies similar to fried eggs with 100-400 µm in diameter Preparation and components of the media to grow mycoplasma are described in detail elsewhere (62- 64). The microbiological culture method is a gold standard to detect every kind of mycoplasma contamination in cell culture without considering the origin and species of mycoplasma. Some strains of Mycoplasma *hyorhinis* cannot cultivate well in this method; however, a certain number of M. *hyorhinis* can grow in mycoplasma cultivation media (65).

The second approved method by the European Pharmacopeia (62) is DNA staining by fluorochromes [such as 4, 6-diamidino-2-phenylindoledihydrochloride (DAPI) and Hoechst 33258 stain]. Although it is practically an easy and rapid test, sometimes interpreting the results is difficult and some experience is definitely necessary. If the condition of cell culture is not good, the DNA staining results will be misinterpreted. To improve the sensitivity and specificity of the direct DNA staining method, using indicator cell lines (e.g. Vero B4, NIH-3T3 or 3T6 cell lines) is helpful. In this case, the method is called indirect DNA staining (29, 66).

Mycoplasma contamination can be detected by polymerase chain reaction (PCR). PCR is easy, sensitive, specific, fast, reliable, efficient and costeffective. The PCR test is based on the detection of 16S rRNA molecules of the most common species of mycoplasma contaminating cell cultures. The specificity of primers in this method should be broad enough to recognize Acholeplasma as well, but narrow enough to prevent amplification of common bacteria which might exist in the PCR reagents (19, 67).

Newly developed methods such as fluorescence in situ hybridization (FISH) or assays based on the detection of adenosine triphosphate (ATP) generation by fluorescence microscopy and luminometer are suggested for mycoplasma, but no published data are available with regard to the sensitivity, specificity, and the accuracy of both assays applied in routine cell culture. However, the speed of these assays is considerable. For example, the results of FISH test can be released in 2 to 3 hours, and the results of luminescence test can be generated within 20 minutes (68).

#### Consequences of mycoplasma contamination in cell culture

Despite the commensal nature of mycoplasmas, their influence on eukaryotic cells and consequently the experimental results cannot be ignored. The overgrowth of mycoplasma can result in loss of the cell culture and irreversible deterioration of the eukaryotic cells. The behavior of mycoplasmas in cell culture is different; therefore, no consistent effects have ever been reported. The activity of arginine deiminase as well as uptake and depletion of the growth medium by mycoplasmas can inhibit the cell proliferation and induce apoptosis in cell lines. Reduction of arginine will result in abnormality of growth rate, decrement of viability, detachment of adherent cells from the cell culture vessel surface, and granulation of cells. Moreover, chromosomal aberration will happen due to the lack of arginine as a major component of the histone in the nucleus (69, 70). Chromosome breakage, multiple translocation events, and numerical chromosome changes are other effects of different species of mycoplasma on cell cultures (71). Exonuclease and endonuclease produced by mycoplasmas are effective in degrading DNAs and RNAs of eukaryotic cells.

The side-effects of mycoplasma contamination on cell cultures are 1. inhibition of proliferation, 2. increment in cell death, 3. fragmentation of DNA and 4) morphological features of apoptosis (72) (Table 3). DNA fragmentation and loss of chromosomal DNA in monocyte cell lines are caused by M. *fermentans*. This cytocidal effect led to the production of non-lipid associated protein fraction (73). The presence of mycoplasmas in cell cultures has different side effects including loss of time, money, valuable cells and misleading publications, besides personal embarrassment and biosafety concerns (40). According to what is mentioned about the consequences of mycoplasma contamination, establishing proper controls and testing cell cultures used in biomedical science is crucial.

#### Elimination of mycoplasma contamination in cell culture

As mentioned above, mycoplasmas cannot be regarded as harmless bystander organisms in cell cultures. Different methods have been developed for treatment of mycoplasma contaminated cell cultures. They are classified as physical (e.g., autoclaving), chemical (e.g., treating with detergent), immunological (e.g., mycoplasma-specific antisera, passage in nude mice, macrophages) and chemotherapeutical (e.g., antibiotics) procedures. Up to now, there is no effectual and adaptable method for mycoplasma eradication in all cases. Elimination of mycoplasmas from cell cultures can be hindered by antibiotic resistance, cytotoxicity of anti-mycoplasma treatments and reduced viability of chronically infected cells (74). The most reliable procedure in mycoplasma elimination is antibiotic administration (9); however, antibiotic resistance in mycoplasmas has occurred. Table 4 shows the findings of researchers at Bionique Testing Laboratories who assessed the incidence of antibiotic resistance in mycoplasmas isolated from infected cell cultures (40).

**Table 4:** Antibiotic resistance of mycoplasma from infected cell cultures


Antibiotic	Resistance
Chloramphenicol	30%
Chlortetracycline	11%
Ciprofloxacin	15%
Erythromycin	98%
Gentamicin	80%
Kanamycin	73%
Lincomycin	28%
Neomycin	86%
Spectinomycin	14%
Streptomycin	88%
Tetracycline	14%
Tylosin	21%


Macrolides, tetracyclines and quinolones are three groups of antibiotics which are shown to be highly active against mycoplasmas (Table 5) (9, 65). There are three different ways to treat the mycoplasma contaminated cells with antibiotics:

1. A. Using quinolones as a single antibiotic compound.
2. B. Application of two different antibiotics such as plasmocin.
3. C. Applying a combination of minocycline (in tetracycline group) and tiamulin (in macrolide group) in alternating cycles with BM-Cyclin.

Various antibiotics with different inhibitory effects on cellular metabolism can be helpful to eliminate mycoplasma contamination. The mechanism of action of macrolides and tetracyclines is inhibiting protein synthesis, but they bind to different subunits of ribosomes. The quinolone inhibits the DNA replication by obstructing bacterial gyrase. In the case of using just one type of antibiotic, it is highly possible that mycoplasmas escape from the inhibitory mechanism or become resistant to it. Insufficient duration or concentration of antibiotic treatment can cause resistance. It is because of surviving the resistant mycoplasmas in the presence of low amount of antibiotics. These resistant mycoplasmas can neutralize the inhibitory mechanism of antibiotic or change its attack site. They can also pump the antibiotic out (18).

**Table 5:** Effective anti-mycoplasma antibiotics


Brand name	Generic name	Antibiotic category
BM-Cyclin	Tiamulin (BM-Cyclin 1)	Macrolide
	Minocycline (BM-Cyclin 2)	Tetracycline
Ciprobay	Ciprofloxacin	Quinolone
Baytril	Enrofloxacin	Quinolone
Zagam	Sparfloxacin	Quinolone
MRA	unknown	Quinolone
Plasmocin	unknown	Tetracycline?
	unknown	Quinolone


The efficiency of antibiotics in elimination of mycoplasmas is between 66 and 85 percent. These percentages include the cultures in which the growth of eukaryotic cells was inhibited, though. Three to 11 percent of cells which are already in a bad condition with a high infection level are lost after antibiotic treatment. However, this event depends on the antibiotic (65).

Elimination of mycoplasmas is usually difficult or unsuccessful due to the resistance of mycoplasmas to antibiotics. It is more successful to passage mycoplasma contaminated cells in nude mice; however, the recovery of cells is not always guaranteed. But when the cells can be collected from subcutaneous tumors in nude mice, the cells are free from mycoplasmas together with a large number of macrophages. Since there is no thymus in nude mice and surely no T-cell dependent immune response, it is possible the macrophages are in charge of the elimination of mycoplasmas. This hypothesis was proved by a brief co-cultivation of mycoplasma contaminated cells with mouse macrophages (75, 76).

Autoclaving the contaminated cell cultures is the best way to get rid of the infections. In the case of valuable cells contaminated by mycoplasmas, autoclave cannot be helpful and an elimination method should be used without harming the eukaryotic cells. Besides the treatment of cell cultures, surfaces, cell culture media and supplements can also be treated by different methods including autoclaving, filtration, exposure to detergents, culture in the presence of 6-methylpurine deoxyriboside, passage through nude mice, and antibiotic treatment (77).

To eradicate mycoplasmas from FBS and trypsin, UV irradiation is more effective than the mentioned methods. In contrast to gamma irradiation, UV irradiation does not harm the serum components. UV irradiation can eliminate any potential contamination in trypsin or FBS (78).

A frequent check up is needed to verify a complete eradication of mycoplasmas. Sometimes mycoplasmas are suppressed and their titer decreases below detection levels of available assays. Thus, a sensitive mycoplasma testing is necessary to detect any reinfection in cell cultures. It is advised to recheck the cells in antibiotic-free medium after at least four to six subculture passages.

## Conclusion

Due to the small size of mycoplasmas and their invisible characteristics, mycoplasmas can spread vastly among the cell cultures. The main sources of mycoplasma contamination vary from personnel to materials and equipments used in cell culture.

The best and the most efficient ways to prevent mycoplasma contamination in cell cultures are following a strict rule for a good aseptic technique and controlling the sources of making aerosols which are the major route for distribution of mycoplasma contaminations.

A quarantine step should be considered to test the new cell lines which are provided from an outside source.

Sensitive and specific tests are necessary to detect mycoplasma contaminations. Performing at least two tests to confirm the mycoplasma contamination in a cell culture is advisable. This is because of the limitations in each detection test. Some strains of M. hyorhinis cannot cultivate well in microbiological culture methods; therefore, other methods such as DNA staining or PCR should be performed in parallel with it to show whether there is any mycoplasma contamination or not. On the other hand, PCR and DNA staining methods cannot distinguish whether mycoplasmas are viable or not. This limitation can affect any decision to continue the treatment of mycoplasma contaminated cell cultures or discarding them. In this case microbiological culture methods can be helpful.

Elimination of mycoplasma is mainly unsuccessful due to unavailability of the antibiotics inside the cells where some of the mycoplasmas hide and escape from treatments. Then, they can expose after a while. The best way to get rid of mycoplasmas is discarding contaminated cells. In the case of materials and supplements for cell culture, it is advised to provide them from companies where trypsin and FBS are decontaminated by a combination of gamma and UV irradiation since mycoplasmas are tiny and can escape through the filter pores when high pressure is applied during filtration.

Since elimination of mycoplasmas from the cells is troublesome, treating mycoplasma contaminated cells by antibiotics is only recommended when the cells are valuable and it is impossible to provide them again.
